# Applying math onto mechanisms: mechanistic knowledge is associated with the use of formal mathematical strategies

**DOI:** 10.1186/s41235-016-0044-1

**Published:** 2017-01-30

**Authors:** Allison S. Liu, Christian D. Schunn

**Affiliations:** 1 Department of Psychology, University of Pittsburgh, Pittsburgh, PA USA; 2 Learning Research and Development Center, University of Pittsburgh, 3939 O’Hara Street, Pittsburgh, PA 15260 USA

**Keywords:** Embodied analogy, Mathematical strategies, Mechanistic knowledge, Proportional reasoning

## Abstract

It is notoriously difficult for people to adaptively apply formal mathematical strategies learned in school to real-world contexts, even when they possess the required mathematical skills. The current study explores whether a problem context’s mechanism can act as an “embodied analogy” onto which abstract mathematical concepts can be applied, leading to more frequent use of formal mathematical strategies. Participants were asked to program a robot to navigate a maze and to create a navigation strategy that would work for differently sized robots. We compared the strategy complexity of participants with high levels of mechanistic knowledge about the robot against participants with low levels of mechanistic knowledge about the robot. Mechanistic knowledge was significantly associated with the frequency and complexity of the mathematical strategies used by participants, suggesting that learning to recognize a problem context’s mechanism may promote independent mathematical problem solving in applied contexts.

## Significance

People typically have a variety of problem-solving strategies available to them for any given mathematics problem. The difficulty lies in selecting the appropriate problem-solving strategy that one learns in classrooms to later, real-world experiences. This is an especially prevalent problem for situations that are not overtly mathematical but would benefit from the use of formal mathematics strategies, as people often default to simpler, more intuitive strategies such as guess-and-check, even when a more formal strategy would be more effective. The current study explores whether an individual’s mechanistic knowledge about a problem situation can influence the strategies that they use to solve the problem. Our findings show that higher levels of mechanistic knowledge are associated with more frequent and complex mathematical strategy use, suggesting that mechanistic knowledge may be one pathway through which adaptive mathematical strategy use can be improved. Encouraging students to break down problem situations into their constituent, mechanistic parts and to think deeply about the mechanistic relationships within a problem could lead to more frequently use of formal mathematical strategies in applied problem contexts.

## Background

Students learn multiple strategies for problem solving during schooling. The challenge is whether they will be able to apply the most appropriate strategy outside of the classroom in real-world contexts, where they are unlikely to receive guidance about how best to approach a problem. This may be particularly difficult when a problem involves mathematics, given the wide range of strategies that are often applicable to a single situation. For example, an algebra problem can be solved using intuitive strategies such as guess-and-check (i.e., inputting and testing unsystematic values in place of the algebraic unknown), which are simple and easy to apply, but can be ineffective in terms of accuracy and time. In contrast, the problem can also be solved using more complex strategies such as rearranging the problem to solve for the algebraic unknown, which, while more complex to apply, are likely faster and more accurate with the requisite mathematical knowledge.

There is evidence that both younger children (e.g., Siegler, Adolph, & Lemaire, 1996) and adults (e.g., LeFevre, Sadesky, & Bisanz, 1996; Lemaire & Reder, 1999) can access and flexibly switch between strategies during math problem solving. However, people also tend to default to simpler mathematical strategies regardless of the problem situation and their pre-existing math knowledge. For example, Nathan and Koedinger (2000) investigated the problem-solving strategies for word problems of high school students who had taken, or were currently, taking Algebra I. Despite having extensive practice with algebra, the majority utilized guess-and-check or work-backwards methods (i.e., reversing the mathematical steps given in a problem to determine the algebraic unknown). While such strategies were somewhat successful (approximately 70% correct) and can provide a preliminary representation of a problem before more abstract concepts are applied, in a number of contexts they can also take more time and be overall less accurate than formal strategies. They are also limited to answers that can be guessed or problems that provide numbers from which students can work backwards (Tabachneck, Koedinger, & Nathan, 1995). The students in Nathan and Koedinger’s (2000) study had the requisite knowledge to access mathematical strategies and were in an overtly mathematical context, but still opted for limited strategies; it is likely that limited strategies are even more common in contexts that do not explicitly call for math but would still benefit from mathematical methods. Given that people already utilize more intuitive strategies, the question is how to support people in recognizing when more complex and formal strategies would be more effective in a problem situation.

### Mechanistic knowledge influencing math use through embodied analogies

Physical manipulatives may be one tool that can encourage the selection of appropriate math strategies for a problem. Most prior studies on physical manipulatives have focused on its effects on mathematical learning rather than strategy use, finding that physical manipulatives can positively influence the learning of mathematics. For example, a meta-analysis of 55 studies by Carbonneau, Marley, and Selig (2013) found that concrete materials were generally better for mathematics learning than abstract mathematics instruction. Physical manipulatives are thought to provide several unique affordances, the most obvious being the opportunity to physically engage with a learning task. Embodied cognition theories suggest that action and perception support higher level cognitive processes (Barsalou, 2008), and perceptual interactions can improve memory and learning (e.g., Glenberg, Gutierrez, & Levin, 2004; Klatzky & Lederman, 2002; Kontra, Lyons, Fischer, & Beilock, 2015). However, given the abstract nature of mathematical concepts (especially at higher levels), it is difficult to directly connect mathematical concepts to physical actions, so physicality per se may not be broadly valuable for problem solving with mathematics. Instead, other affordances may play a stronger role. Furthermore, other studies have found that emphasizing the physical aspects of a manipulative can make students focus too strongly on the manipulative itself, distracting students from learning the abstract concept it is meant to represent (see Uttal, O’Doherty, Newland, Hand, & DeLoache, 2009).

We propose that focusing on the physical object can actually be helpful for learning math strategies, so long as the physical manipulative can be used as an “embodied analogy.” The individual parts of the manipulative become places where number values can be applied, and the physical relationships between those parts can support discoveries about how those numerical values interact, providing a concrete visualization of mathematical principles. Consider, for example, the use of a toy car to practice proportional reasoning. While focusing on the toy car may be distracting if students think of it as play rather than mathematics, students may also be able to discover the regular mechanistic relationship between wheel rotations and distance traveled by interacting with the toy car. Having recognized that these parts are related in this way, students can then apply quantitative values to each part, see how these numbers affect one another, and see a concrete example of proportional reasoning at work. It is when these mechanistic relationships are irregular, too abstract, or lost because of extraneous physical details that the usefulness of a manipulative as an embodied analogy would be diminished. For example, a manipulative such as an abacus would be more difficult to use as an embodied analogy, as the mechanistic relationships between the parts (i.e., the abacus beads representing different place values) are more abstract and harder to determine without explicit instruction. Thus, students would be less likely to discover such relationships on their own and be able to apply quantitative values to it (though, with instruction about what each part represents and how they relate, then it is expected to be equally effective). It is possible that routine use of embodied analogy manipulatives could train students to break down problem contexts into their constituent parts and recognize whether regular relationships exist between them, thus allowing them to see whether formal mathematics can be used in that problem. The use of manipulatives that would be found in real-world contexts can further prepare students to apply math principles in less math-overt situations, potentially fostering the use of formal math strategies in later applied contexts.

In order to use a physical manipulative as an embodied analogy that supports mathematical use, a student would need to understand both the mechanistically relevant parts of the manipulative (on which numerical values can be meaningfully applied; e.g., the toy car’s wheels), as well as how those different parts relate (to understand the relationships between the numerical values; e.g., the relationship between the toy car’s wheel rotations and distance traveled). This is supported by research that has distinguished two types of visuospatial representations that appear to affect mathematical problem solving: pictorial representations, which describe the specific people, places, or objects in a problem, and schematic representations, which depict the spatial relations involved in a problem. The use of schematic representations positively correlates with mathematical problem-solving success, while the use of pictorial representations negatively correlates with mathematical problem-solving success (Hegarty & Kozhevnikov, 1999; van Garderen & Montague, 2003). As a more extreme performance contrast, students with learning disabilities use more pictorial representations than gifted students (van Garderen & Montague, 2003). This suggests that people must have knowledge of both the parts of a problem context and their relations for mathematical problem solving to be improved.

There is also evidence that thinking about a manipulative’s parts and relations while learning can influence later mathematics performance. Silk (2011) taught proportional reasoning concepts to middle-school students using a “robot dancing” exercise. Student groups were asked to synchronize the movements of differently sized robots into a choreographed dance. Half of the student groups were encouraged to use mathematics to model their intuitions about how the robot worked (i.e., to think *mechanistically* about mathematics and the robot’s parts), while other groups were encouraged to use mathematics to calculate the input values needed to get desired output values for the dancing task (i.e., to think *calculationally* about mathematics and the robot). The mechanistic student groups significantly improved on a proportional reasoning test compared to the calculational student groups. Furthermore, interviews showed that all four of the mechanistic groups transferred their strategies from one robotics task to another robotics task, compared to only one of the four calculational groups, suggesting that thinking mechanistically during learning can positively affect mathematical problem solving and close transfer.

Still, little work has investigated how mechanistic knowledge can influence the likelihood of using formal mathematical strategies in more complex problems that are not clearly mathematical in nature, or when a person already has the necessary mathematical knowledge to solve the problem. Having high mechanistic knowledge of a problem may make the structural components of a problem and their connections clearer, which may make it easier to use it as an embodied analogy and to apply formal mathematics to the problem when appropriate. Alternatively, high mechanistic knowledge may instead overemphasize extraneous details that distract from the relevant mechanistic parts that can be used for an embodied analogy (Belenky & Schalk, 2014; Harp & Mayer, 1998; Kaminski, Sloutsky, & Heckler, 2009) or inhibit transfer to novel situations (e.g., Sloutsky, Kaminski, & Heckler, 2005; Son & Goldstone, 2009).

### The current study

In the current study, we begin exploring embodied analogies by investigating whether mechanistic knowledge influences the frequency or generalizability of mathematical strategies during an applied problem-solving task. Notably, we test participants who have the required knowledge to solve the task through formal mathematics; the question is whether they will do so when the task does not explicitly call for math. Thus, our participants are students from a fairly selective college that requires above average quantitative Scholastic Assessment Test (SAT) scores for admission.

We chose a task that could be solved with relatively basic mathematics for college students. In particular, the task involves determining the scalar or ratio relationship between two quantities. Since the quantitative SAT contains many questions about proportional and algebraic reasoning, the participants, who all did relatively well on this test, possessed sufficient mathematical ability to complete the given task.

In the task, we ask participants to program a LEGO NXT robot to navigate through a maze. The robot’s mechanism involves three parts: the robot’s program, motors, and wheels. The program (which used a C-based language called ROBOTC; www.robotc.net) consists of commands that tell the robot which direction to move and the number of times to rotate its motors (through commands in the form of “*direction* (*number of motor rotations*)”). When the program is run through the robot’s interface, the robot’s motors rotate the number of times designated in the commands, causing the robot’s wheels to rotate, which causes the robot to move. The number of motor rotations, the number of wheel rotations, and the distance that the robot travels are proportionally related: one motor rotation will always lead to the same number of wheel rotations, which will always lead to the same distance traveled by the robot. This insight is a relatively straightforward discovery from numbers produced through experimentation, and middle-school-aged children regularly made this discovery in earlier work with this task (Silk, 2011).

In addition to navigating the robot through the maze, participants are also asked to create a generalizable strategy that could navigate differently sized robots through the same maze. Although nonmathematical strategies (i.e., guessing and checking) could be used to navigate the provided robot through the maze, formal mathematics (i.e., proportional reasoning) are a necessary component of strategies that would generalize across differently sized robots. We investigate the role of mechanistic knowledge by comparing the strategies of participants who completed a short task emphasizing the robot’s motor-wheel relationship (High-Mechanistic condition) against the strategies of participants who were not given this task (Low-Mechanistic condition).

However, we observe considerable migration across conditions: while most participants in the original High-Mechanistic condition are able to accurately describe the robot’s mechanism (71%), many participants from the original Low-Mechanistic condition are also able to do so (54%). Thus, we recategorize our High-Mechanistic and Low-Mechanistic groups based on participants’ Original Condition and their performance on a mechanistic knowledge assessment at the end of the study (the Mechanistic Assessment) to take both the task emphasizing motor-wheel relations and any mechanistic knowledge discovered individually into account. These analyses are reported in the “Results” section. In addition, we provide results using the Original Condition assignment, as well as group assignment based only on the Mechanistic Assessment (ignoring Original Condition), for comparison in Table 1, noting that results for all three assignment methods trend in the same direction.

**Table 1:** Summary of results comparing High-Mechanistic and Low-Mechanistic participants defined by Original Condition, Mechanistic Assessment, and Matched Original Condition and Mechanistic Assessment

Task	Original Condition (*N* = 50)	Mechanistic Assessment (*N* = 50)	Matched (*N* = 29)
Navigation strategy	High = Low	High > Low	High > Low
MRMQ	High = Low	High > Low	High > Low
Memory drawings	Wheels: High = Low Motors: High > Low Screens: High = Low	Wheels: High = Low Motors: High = Low Screens: High = Low	Wheels: High = Low Motors: High > Low Screens: High = Low
Significant predictors of strategy	MRMQ: *β* = 0.37 Mastery goals: *β* = −0.45	Group: *β* = 0.35	(Forward) Group: *β* = 0.61 (Backward) Group: *β* = 0.69 Mastery goals: *β* = −0.42 Performance goals: *β* = 0.48

*MRMQ* Math in Robot Motion Questionnaire

Using the recategorized groups, we hypothesize that the High-Mechanistic group will use more complex and accurate mathematical strategies than the Low-Mechanistic group, because they are more likely to consider the mechanistic relationship between the robot’s motors and wheels and, therefore, discover the proportional relationships between motor rotations, wheel rotations, and distance traveled.

## Method

### Participants

Participants consisted of 50 undergraduate students recruited through the psychology department’s subject pool. To avoid ceiling effects, students majoring in robotics-related or math-heavy majors were not eligible to participate in the study because they (unlike much of the general population) must reason mathematically about many situations on a regular basis. Originally, 24 participants were randomly assigned to the High-Mechanistic condition and 26 participants were randomly assigned to the Low-Mechanistic condition. However, participants were recategorized into a High-Mechanistic (*n* = 17) group and a Low-Mechanistic groups (*n* = 12) using criteria described in the Mechanistic Assessment section below, while 21 participants were excluded from analyses (though analyses on the full sample based on Original Condition or Mechanistic Assessment are shown in Table 1). For participants who reported their quantitative SAT scores, scores did not significantly differ between the High-Mechanistic (*M* = 647 = 85th percentile, *SD* = 79) group, the Low-Mechanistic (*M* = 639 = 83rd percentile, *SD* = 86) group, and the excluded participants (*M* = 618 = 79th percentile, *SD* = 101), though five High-Mechanistic group, five Low-Mechanistic group, and six excluded participants did not report their SAT scores. Average reported SAT scores were high enough to suggest that participants likely had the proportional reasoning knowledge to complete the task, even though their majors did not require frequent use of mathematics.

### Materials

#### Mechanistic Manipulation

**Fig. 1:**
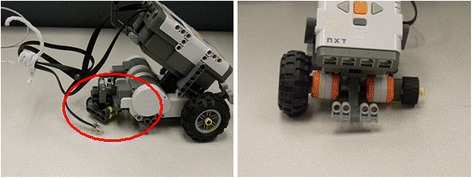
The two robots used in the Mechanistic Manipulation. *Left*, the USB cord connecting the robot’s right motor to the robot’s brick was disconnected. *Right*, the robot’s two wheels were mismatched in size

Participants in the original High-Mechanistic condition were shown (but did not physically interact with) two defective physical LEGO NXT robots and asked to predict whether the robot would be able to move forward in a straight line. On the first robot (Fig. 1, left), the cord attaching the robot’s brick (where the robot’s programs and commands are stored) to the robot’s motors was disconnected to emphasize the relationship between the robot’s commands and wheel movements via motor rotations. The second robot (Fig. 1, right) had two mismatched wheels (one large wheel and one small wheel) to emphasize the relationship between the robot’s wheel size and movement distance. An experimenter ran each robot to test participants’ predictions and show that the robots would not run properly. The experimenter also explained the cause of the robots’ errors (specifically that the brick was disconnected from the motors, and that the wheels were different sizes). After seeing both robots, participants were asked to describe the process through which the robot goes to move forward, starting from the moment a program is downloaded into the robot; if participants’ responses contained errors, the experimenter corrected them before moving on to the next task. These explanations did not provide explicit information about the mathematical relationships between the parts of the robot. The manipulation took approximately 5 min to complete. Because of the manipulation’s short duration, the original Low-Mechanistic condition did not experience a parallel manipulation to avoid introducing potential confounds with a control task.

#### Mechanistic Assessment

At the end of the experiment, participants were asked two open-ended questions about how the robot functioned to assess their mechanistic knowledge of the robot: “Please explain the process that the robot goes through to move, starting from its motor rotating” and “Please draw a diagram of the process.” Participants were categorized into the High-Mechanistic group if they had received the Mechanistic Manipulation *and* indicated in at least one of the two questions that the robot’s mechanism included both motors and wheels, and that the motor rotations caused the robot’s wheels to rotate (i.e., they recognized the fundamental mechanism that connects the parts of the robot). Participants were categorized into the Low-Mechanistic group if they had not received the Mechanistic Manipulation *and* had not accurately answered either of the Mechanistic Assessment questions. Participants who either received the Mechanistic Manipulation but did not accurately answer either of the Mechanistic Assessment questions or did not receive the Mechanistic Manipulation but accurately answered one of the two Mechanistic Assessment questions (i.e., participants who migrated out of condition) were excluded from analyses. We chose these criteria to define our groups for analysis because participants who received the Mechanistic Manipulation and were able to correctly answer one of the Mechanistic Assessment questions likely had more robust mechanistic knowledge of the robot compared to participants who did not receive or discover any information about the mechanism. It is also doubtful that participants’ responses to the Mechanistic Assessment reflect prior mechanistic knowledge and instead represent mechanistic knowledge that was learned from the Mechanistic Manipulation or developed during the experiment’s duration because no participants reported post-childhood experience with nonrobotic LEGO and only two participants in the excluded group reported high-school experiences with robotics. Thus, while we expect the Mechanistic Manipulation to be the primary method increasing mechanistic understanding, we are able to account for participants who migrated across conditions (7 originally in the High-Mechanistic condition, 14 originally in the Low-Mechanistic condition) by including the Mechanistic Assessment responses in group assignment. Two independent coders rated responses to the assessment (Kappa = 1.0). Examples of typical High-Mechanistic and Low-Mechanistic responses are shown in Table 2.

**Table 2:** Examples of High-Mechanistic and Low-Mechanistic answers on the Mechanistic Assessment

	Example 1	Example 2
High-Mechanistic	“First, you create the path on the computer that the robot will go, then, plugging in the USB, I downloaded the software to the box on the robot. From there, once I press run, the wires connected to the box take that info from the box to the motor of the robot, which then moves the wheels.”	“Instructions given from the computer travel to the body which sends them to the motor. The motor turns which then turns the wheels.”
Low-Mechanistic	“When going forward and backward, the robot doesn’t move straight. When going left and right, it spins in the desired direction.”	“The motor gets its instructors from the computer which tells what direction to go and for how long to go in that direction.”

#### Maze Navigation Task

**Fig. 2:**
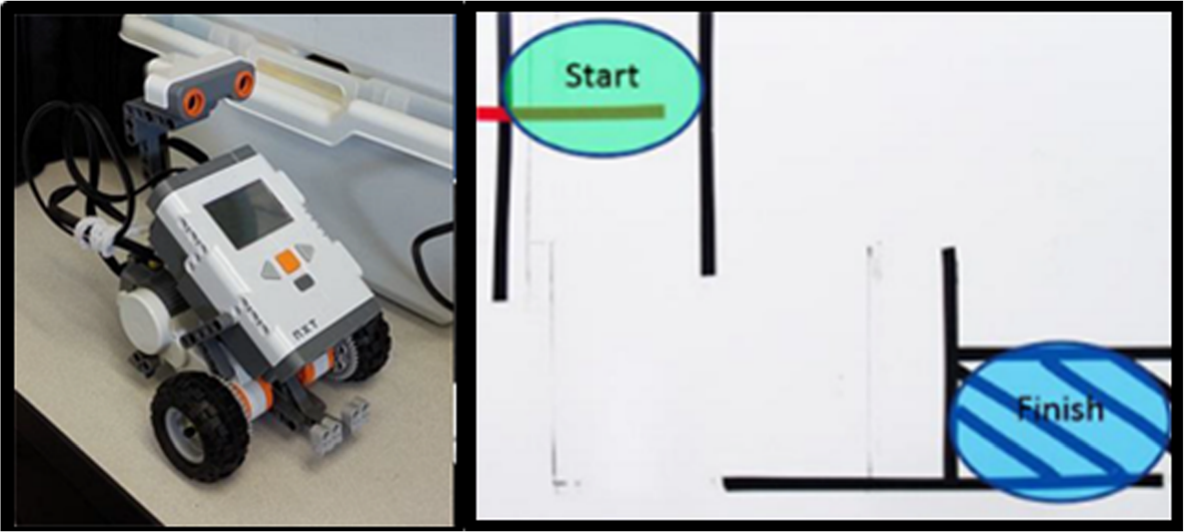
The robot (*left*) and a top-down picture of the maze used in the Maze Navigation Task. *Thick black lines* represent barriers that should not be crossed. The *thick red line* in the *green start oval* indicates the starting position, behind which the robot must be placed. The *blue finish oval* indicates the ending position where the robot should be navigated

Participants learned to program a physical LEGO NXT robot (see Fig. 2, left) using ROBOTC, a C-based language that was designed to simplify robot movement programming. Four commands given to participants that were critical for this task told the robot which direction to move and the number of times to rotate its motor during each movement (e.g., forward (100), backward (150), turnRight (50), turnLeft (30)).

Participants were given 30 min to navigate their robot through a physical maze on a 134-cm × 148-cm whiteboard placed on the ground (see Fig. 2, right). Participants were provided with a tape measure to measure the robot or maze, though there was no requirement to use the tool. Participants were also asked to create a navigational strategy that subsequent participants could use to complete the same task (though possibly with differently sized robots) and were told that guess-and-check strategies were generally inefficient for this purpose. After creating their initial strategy, participants were granted an additional 30 min to revise their strategy, with a reminder that their strategy should generalize to robots with differently sized wheels, and they were given access to a set of smaller robot wheels for testing purposes. Two independent raters coded participants’ final strategies into one of four types based on the type of strategy used: Guessing, Plausible Guesstimation, Specific Proportional, and General Proportional (definitions and examples shown in Table 3), with Kappa = 1.0. The first two strategy types do not explicitly use mathematics, though prior research suggests that the Plausible Guesstimation strategy is a foundation upon which more sophisticated mathematical strategies can be built (Nhouyvanisvong, 1999). The latter two mathematical strategy types are both relevant to the task, but only a General Proportional strategy can fully solve the task given to the participants. Note that the width of the maze corridors is significantly larger than the width of the robot, so more than one solution at the minor detail level is possible for the base path through the maze.

**Table 3:** Codes used for the Maze Navigation Task

Code	Description	Example
Non-math: Guessing	Participant created a guess-and-check strategy with no clear basis for guessed numbers	“Go straight direction, forward (100). turnLeft (28), 28 is still too large to turn, 100 is too long. Go straight like first step, but the length is a little shorter, forward (100).”
Non-math: Plausible Guesstimation	Participant created a guess-and-check strategy; guessed numbers were estimated using some situational basis	“Guess + test was my main strategy. After I learned that it took the robot 150 (approx.) motor rotations to go one straight stretch of the maze + 30 (approx.) motor rotations to make a turn in the maze, I just entered in the numbers until finally the robot got through the maze.”
Math: Specific Proportional	Participant created a strategy utilizing proportional reasoning; values were specific to their robot	“It is 0.1 in. per motor-rotation. […] Measure the distance for each straight trait which is divided by 0.1 to get the number of motor-rotations for each straight trait.”
Math: General Proportional	Participant created a strategy utilizing proportional reasoning that could be generalized to other robots	“Start off with a given value for motor rotations (*R*1) and measure the distance the robot travelled for that number of rotations (*D*1). Measure the distance you would like the robot to travel to reach its intended destination (*D*2). Calculate the number of rotations it will take the robot to travel this distance using the formula *R*1/*R*2 = *D*1/*D*2.”

We purposefully designed the navigation portion of the task to be fairly easy because we wanted all participants to progress far enough to be able to have time to focus on the creation of their strategies. Furthermore, the outcome variable of interest was not whether participants could successfully navigate through the maze, but whether their solution strategy could be generalized. To create a generalizable strategy, participants must have uncovered the simple proportional relationship between wheel size and distance per motor rotation, and between wheel size and angle turned per motor rotation. Participants could conceptualize these relationships in absolute/functional terms (by reasoning about the multipliers between wheel diameter and centimeters per motor rotation or angle per motor rotation) or in relative/scaling terms (by reasoning about the scaling constants between the tested robots and other robots).

It is important to note that while the parts of the robot’s mechanism (i.e., the wheels and motors) are also the parts of the robot that can be quantified in a formal mathematical strategy, high mechanistic knowledge of the robot’s details is not logically necessary for the creation of a mathematical formula. For example, participants could figure out a pattern between the numbers inputted into the program and the distance traveled by the robot and derive a formula from that pattern, without ever determining the relation between the motor and the wheels of the robot.

#### Math in Robot Motion Questionnaire (MRMQ)

To more directly test knowledge about the mathematical relationships involved in solving robot motion problems, this questionnaire consisted of eight open-ended questions about the quantitative relationships between the robot’s motor rotations, wheel rotations, and distances (e.g., “Are the number of wheel rotations related to the distance that the robot moves forward?”). Each response was scored for the number of accurate mathematical relationships included in the answer, such that a higher score signified greater quantitative understanding (with a maximum score of 16 points). Cronbach’s alpha (*α*) for this questionnaire was only 0.64, suggesting that an understanding of each mathematical relationship was not strongly dependent on one another (i.e., there were separate insights rather than just one overall insight). The overall alpha is still sufficiently high to justify analysis as one overall construct.

#### Robot-drawing Tasks

Participants were asked to draw the robot that they had programmed from memory. These drawings from memory provided a measure of detail in participants’ representations of the robot. For the memory drawing, the robot was taken away, and participants drew on a blank sheet of 8.5-in. × 11-in. paper. They were specifically told to include the most important parts of the robot in their drawing. A second drawing task, designed to control for drawing ability across participants, was given with the same instructions, except participants could look at and manipulate the robot as a reference while they drew. To distinguish between proportionally relevant and proportionally irrelevant parts of the robot’s mechanism, two raters coded for the number of accurately drawn wheels and the number of motors included in the drawings (mechanistically and proportionally relevant features), and whether the drawing included a detailed depiction of the robot’s screen (a mechanistically relevant but proportionally irrelevant feature) (Kappa = 1.0).

#### Paper Folding Test

This test is associated with the ability to perform dynamic intrinsic spatial transformations (Ekstrom, French, Harman, & Dermen, 1976; Shepard & Feng, 1972). It is correlated with the ability to perform complex animation tasks (Hegarty, Kozhevnikov, Gero, & Tversky, 1999), which may be a critical factor in turning mechanistic understanding of robot movements into mathematical patterns. This measure is also useful in ruling out a confound of spatial ability as the underlying variable between mechanism understanding and mathematical strategy use. A series of pictures depicts one to three folds made in a piece of paper, and the final picture shows a hole punched into the paper. Participants selected which of five options illustrated the reopened piece of paper. The test consisted of two parts with 10 questions each (*α* = 0.84).

#### Motivation questionnaire

As a control variable, participants answered nine questions about their level of motivation during the Maze Navigation Task, building upon theories and measures of engagement (the Intrinsic Motivation Inventory, e.g., Ryan, 1982) and achievement goals (Elliot & Church, 1997). Three questions asked about the participants’ level of engagement (*α* = 0.91), three questions asked about the participants’ level of performance-approach goals (*α* = 0.86), and three questions asked about participants’ level of mastery-approach goals (*α* = 0.80). Participants were asked to rate their agreement with each statement on a scale of 1 (Strongly disagree) to 7 (Strongly agree).

### Procedure

Participants gave informed consent before starting the experiment. Participants first completed the Paper Folding Test. Next, an experimenter gave a brief, verbal introduction to the LEGO NXT robot. The introduction informed participants that they would be typing commands into the robot’s program that would tell the robot which direction to move and how many times to turn its motor, which would cause the robot to move. While the introduction did not explain the robot’s full mechanism (i.e., that the motors rotate, causing the wheels to rotate, causing the robot to move), it implied that the robot’s motors were connected to the robot’s wheels (as the wheels are the only mechanism through which the robot can move). Thus, differences between participants could not be fundamental misunderstandings about the source of movement, and instead were expected to be at the level of understanding the motor-to-movement relationship. Participants in the original High-Mechanistic condition completed the additional Mechanistic Manipulation, while participants in the original Low-Mechanistic condition moved on immediately to the next task.

All participants then began the Maze Navigation Task, which was introduced as a programming task and included basic programming instructions. After 30 min, participants were given an additional 30 min to revise their initial strategy while working with a robot with smaller-sized wheels. At the end, participants were given the Robot-drawing and Control-drawing Tasks, as well as the Motivation, Mechanistic Assessment, and Math in Robot Motion Questionnaires.

## Results

### Maze strategies

**Fig. 3:**
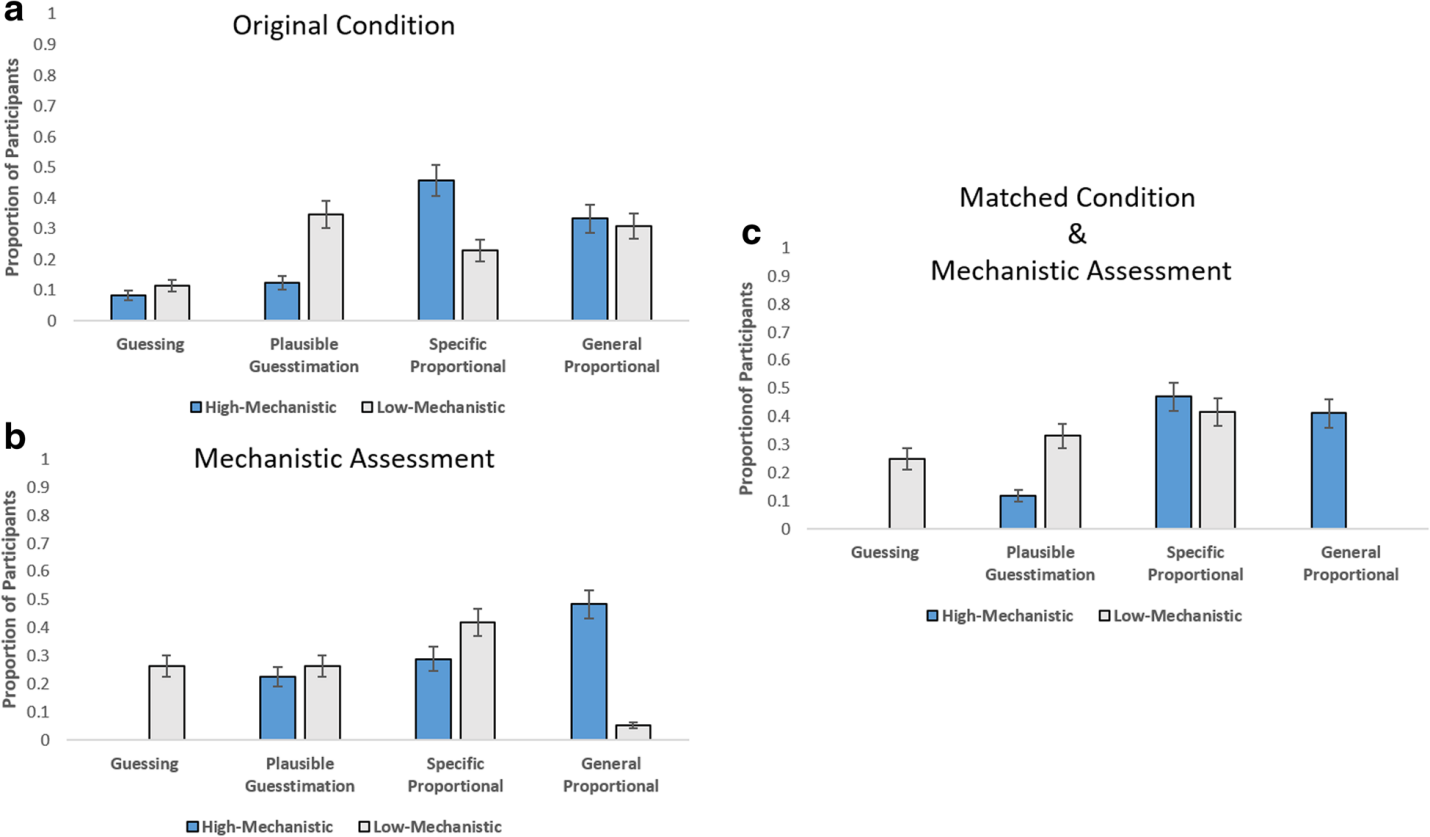
Proportion of participants using each Maze Navigation Task strategy type based on (**a**) their Original Condition assignment, (**b**) their Mechanistic Assessment performance, and (**c**) their recategorized Mechanistic group based on Original Condition and Mechanistic Assessment performance

Figure 3 shows the proportion of participants who created each type of final strategy based on their (1) Original Condition, (2) their Mechanistic Assessment performance, and (3) their recategorized Mechanistic group (based on matching their Original Condition with their Mechanistic Assessment performance). We examined strategy complexity between the High-Mechanistic and Low-Mechanistic groups, with Guessing being the least complex strategy possible, and General Proportional being the most complex strategy possible. As hypothesized, the High-Mechanistic group (mean rank = 19.0) was significantly more likely to create complex strategies than the Low-Mechanistic group (mean rank = 9.33), *U* = 34.0, *p* = 0.002, *r* = −0.59. In general, participants with higher mechanistic knowledge created proportional strategies that were more mathematically complex (and consequently more accurate), while participants with lower mechanistic knowledge relied primarily on estimation or limited proportional strategies. In general, participants with higher mechanistic knowledge created proportional strategies that were more mathematically complex (and consequently more accurate), while participants with lower mechanistic knowledge relied primarily on estimation or limited proportional strategies.

### Math in Robot Motion Questionnaire

The High-Mechanistic group (*M* = 6.12, *SD* = 2.67) had significantly higher scores on the MRMQ than the Low-Mechanistic group (*M* = 3.75, *SD* = 2.45), *t*(27) = −2.43, *p* = 0.02, *d* = 0.92, showing a greater understanding of the quantitative relationships that exist within the robot. Participants’ scores on the questionnaire also positively correlated with the complexity of their final maze strategy, *r*(29) = 0.61, *p* < 0.001, indicating that participants who created more mathematically complex strategies in the Maze Navigation Task were those who possessed greater understanding of the quantitative relationships within the robot, not simply participants who chose to use this understanding in their solutions.

### Robot-drawing Tasks

As expected, there were no differences between the number of accurately drawn wheels or motors in the control drawings (when participants were able to use the robot as a reference while they drew), showing that there were no differences in drawing ability between the High-Mechanistic and Low-Mechanistic groups. For the memory drawings, both groups tended to draw all three wheels (High-Mechanistic mean rank = 15.09; Low-Mechanistic mean rank = 14.88), *U* = 100.5, *p* = 0.95, *r* = −0.02, and to include the key screen details in their drawings (High-Mechanistic: 83% inclusion; Low-Mechanistic: 88% inclusion), *X* ^*2*^(1, *N* = 29) = 0.14, *p* = 0.56. However, the groups significantly differed in the number of motors included in their memory drawings, *U* = 32.0, *p* = 0.001, *r* = −0.62. The High-Mechanistic group (mean rank = 19.1) included significantly more motors in their drawings than the Low-Mechanistic group (mean rank = 9.17). Thus, while the groups showed equivalent encoding for the more visually salient parts of the robot (i.e., the screen and wheels), there were differences for the less visually salient, but still mechanistically relevant, motors, corroborating our assumption that the High-Mechanistic group possessed more mechanistic knowledge about the robot than the Low-Mechanistic group.

Table 1 shows that there were no significant differences between the number of accurate motors for the High- and Low-Mechanistic groups based on Mechanistic Assessment only. In this case, the average for both groups show that both groups generally included at least one motor in their drawing, suggesting that they had the sufficient mechanistic knowledge about the robot’s wheels and motors that would be needed for the robot to be used as an embodied analogy. Thus, the lack of difference between the High- and Low-Mechanistic groups based on Mechanistic Assignment does not weaken the findings relating mechanistic knowledge and strategy use.

### Controlling for individual differences

To determine whether the relationship between Mechanistic group and Maze Navigation Task strategies was explained by individual differences in spatial ability, motivation, robot math understanding, or encoded perceptual details, multiple linear regression was used to develop a model predicting participants’ used math strategy from their group, Paper Folding Test score, average performance goal rating, average mastery goal rating, average interest rating, MRMQ score, and number of motors included in their memory drawings.

Because our reduced sample (*N* = 29) would likely produce an unstable model if fully saturated, we instead conducted two regressions using forward selection and backward elimination with an entry threshold of *p* = 0.05 and a removal threshold of *p* = 0.1. Forward selection settled on a model in which Mechanistic group was the only significant predictor of maze strategy complexity (*β* = 0.61, *p* < 0.001). Backward elimination settled on a model in which Mechanistic group (*β* = 0.69, *p* < 0.001), mastery goal rating (*β* = −0.42, *p* = 0.03), and performance goal rating (*β* = 0.48, *p* = 0.01) were significant predictors. The forward and backward predictor models were able to account for 37% (*F*(1, 27) = 15.9, *p* < 0.001, *R* ^2^ = 0.37) and 52% (*F*(3, 25) = 9.07, *p* < 0.001, *R* ^2^ = 0.52) of the variance in Maze Navigation Task strategy complexity, respectively. Table 4 shows the zero-order and partial correlations for the reduced participant sample described above (*N* = 29), as well as the fully saturated but poorly fitted model using the original participant sample (*N* = 50), and Table 1 summarizes the significant predictors found with each assignment type (Original Condition, Mechanistic Assessment, and Matched Original Condition and Mechanistic Assessment). Note that the Paper Folding Test, which typically correlates with tests of general intelligence, (e.g., Visser, Ashton, & Vernon, 2006), did not significantly predict strategy choice in any of our models, suggesting that pre-existing individual differences in intelligence are not driving the relationship seen between mechanistic knowledge and strategy choice. Instead, mechanistic knowledge acts as a unique and consistent predictor of strategy.

**Table 4:** Maze Navigation Task strategies related to individual difference measures for the reduced (*N* = 29) and full (*N* = 50) participant sample

Variable	Reduced sample		Full sample	
	*r*	Partial *r*	*r*	Partial *r*
Original Condition	**-**	**-**	0.16	0.13
Mechanistic group	0.61*	0.52*	0.51*	0.29**
Paper Folding Test	0.10	0.03	0.23**	0.13
Interest	0.32*	0.05	0.26*	0.11
Mastery	0.12	−0.40**	0.09	−0.20
Performance	0.32*	0.48*	0.16	0.20
MRMQ	0.41*	0.25	0.38*	−0.07
Drawing (motors)	0.49*	0.02	0.14	0.20

*p < 0.05, **p < 0.07. *MRMQ* Math in Robot Motion Questionnaire

### Discussion

Students are taught a variety of mathematical strategies during schooling that they must learn to appropriately apply to real-world contexts. However, limited intuitive strategies are often used in contexts that are overtly mathematical and even more so in contexts that are not, even when more formal strategies would be more effective and one has the requisite knowledge to apply the said strategies. The current study investigated whether physical manipulatives can potentially improve students’ strategy selection through embodied analogies; focusing participants’ attention on the mechanistic parts and relationships that exist within a manipulative may allow participants to see that numeric values can be applied to these mechanistic parts, thus supporting the creation of formal mathematical strategies (in this case, a formal math formula representing the proportional relationship between the robot’s motor rotations, wheel rotations, and distance traveled). High-Mechanistic participants were significantly more likely to use complex mathematical strategies to solve the Maze Navigation Task than Low-Mechanistic participants. In addition, the MRMQ results showed that High-Mechanistic participants had a greater understanding of the mathematical relationships within the robot, suggesting that mechanistic knowledge may help drive the use of mathematics in an applied context.

Our results support previous findings that mechanistic knowledge can be useful in scaffolding people toward mathematical strategies. Bechtel and Abrahamsen (2005) note that there are two primary ways in which people decompose mechanisms: structurally, with the focus on component parts, or functionally, with the focus on component operations. Thinking in terms of causal processes, as is done when breaking down mechanisms functionally, has been argued to be more beneficial for mathematical thinking (Schwank, 1999). In the current study, the High-Mechanistic participants had sufficient information about both the component parts of the robot’s mechanism (as High-Mechanistic participants, on average, included a large number of wheels and motors in their drawings), as well as the component operations between the robot’s motors and wheels (as shown by their responses on the Mechanistic Assessment), which would lead to better strategies based on this structural/functional divide.

Mechanistic knowledge may also foster mathematical strategies by pushing learners to think about problems at a less superficial level. For example, Russ, Coffey, Hammer, and Hutchison (2009) described a case study in which a second-grade student began building her knowledge of a science problem by exploring naïve mechanistic explanations. When the student’s teacher pushed for a correct answer instead of pursuing the student’s attempts at understanding the mechanism, the student resorted to using textbook terms that she did not fully understand. Consequently, Russ recommended that mechanisms be considered when assessing students’ scientific understanding. Indeed, in many natural sciences, explanations must include an explanation of mechanisms to be satisfactory (Machamer, Darden, & Craver, 2000), and others have advocated for mechanistic understanding within the social sciences as well (see Hedström & Ylikoski, 2010 for a review). Our findings suggest that mechanistic knowledge may be equally useful in mathematics. Providing people with information on the context’s mechanisms may push them toward mathematical problem-solving strategies.

### Limitations

The largest limitation of the current study was that a substantial number of participants migrated out of their Original Condition (i.e., those who were originally in the High-Mechanistic condition were unable to accurately answer the Mechanistic Assessment, and vice versa). It is possible that different recategorization criteria for the “High-Mechanistic” and “Low-Mechanistic” groups could impact the results found. In the current study’s analyses, we used relatively conservative criteria based on participants’ originally assigned condition and their performance on the Mechanistic Assessment, but we also found similar results using different sets of criteria. For example, High-Mechanistic participants still created significantly more complex strategies than Low-Mechanistic participants when: the High-Mechanistic group included participants who were in the original High-Mechanistic condition and answered both Mechanistic Assessment questions correctly, while the Low-Mechanistic group included participants who were in the original Low-Mechanistic condition and answered 0–1 of the Mechanistic Assessment questions correctly; when the High-Mechanistic group included participants who were in the original High-Mechanistic condition and answered both Mechanistic Assessment questions correctly, while the Low-Mechanistic group included all other participants; and (marginally significant) when the High-Mechanistic group included participants who were in the original High-Mechanistic condition and answered at least one Mechanistic Assessment question correctly, while the Low-Mechanistic group included all participants originally in the Low-Mechanistic condition. Thus, mechanistic knowledge appears to have a consistent effect on the use of mathematical strategies, regardless of the way that the sample is split, meaning that embodied analogies may be a plausible intervention for improving the quantity and quality of people’s mathematical strategy use. A separate question is how robust these findings are. As seen in Table 1, results varied slightly depending on the assignment type used (i.e., Original Condition, Mechanistic Assessment, Matched). This suggests that the strength of this intervention likely depends on the situation, and future studies should investigate how robust the association between mechanistic understanding and math strategy use is under different circumstances, and what can be done to strengthen it.

The recategorization criteria used in the current study also excluded a relatively large number of participants from analyses, which may have biased results if these participants were meaningfully different from participants in the High-Mechanistic and/or Low-Mechanistic groups. Participants’ SAT scores suggest that there were no significant differences between the groups’ baseline math abilities (though some participants did not report their scores). Furthermore, when these excluded participants were included in analyses as a third unitary group or as two separate groups, their average scores consistently sat between the High-Mechanistic and Low-Mechanistic groups’ scores for all outcome measures (except for the number of wheels included in memory drawings, which was not significantly different from either group). This implies that these excluded participants possessed some mechanistic knowledge, as they were either introduced to the robot’s mechanism through the Mechanistic Manipulation or discovered the mechanism themselves during the Maze Navigation Task, but that this knowledge was more fragile than the High-Mechanistic group whose mechanistic knowledge was reinforced by both the Mechanistic Manipulation and their experiences during the Maze Navigation Task.

Although we argue that the Mechanistic Assessment measured participants’ level of mechanistic knowledge of the robot, it is possible that the assessment’s questions target a different construct. For example, the questions may be measuring the importance that participants place on their existing mechanistic knowledge (i.e., whether they considered the robot’s motor and wheels important enough to include in their responses). However, even if the assessment did not measure mechanistic knowledge per se, it is still measuring how participants think about the robot, suggesting that readily accessing mechanistic knowledge can influence the use of existing math knowledge.

### Future directions: the relationships between physicality, mechanistic knowledge, and mathematics strategies

The current study is an initial investigation into the use of physical manipulatives as an embodied analogy onto which math concepts can be applied, via mechanistic knowledge about the manipulative’s parts and relationships. While we show evidence that mechanistic knowledge relates to math outcomes (in the form of mathematical strategy use), we could not fully explore the relationship between physicality and mechanistic knowledge, as our study only used a physical manipulative. The effect of mechanistic knowledge may vary depending on the amount of perceptual detail that is encoded from the problem situation. For example, effective use of mechanistic knowledge may require sufficient knowledge about the problem situation’s component parts, which a physical or concrete environment may provide, but a reduced virtual environment may not; thus, the mechanistic knowledge effect may have diminished if our study had used virtual robots instead of physical robots. Future studies could investigate this relationship, which would provide further information about the effects of physical manipulatives on problem-solving strategies, as well as the affordances of physical manipulatives. Furthermore, we did not directly examine how participants were applying mathematics onto the physical manipulative. Future studies could investigate this more explicitly (e.g., through talk-aloud protocols or questionnaires about strategy development during the problem task).

The existence of embodied analogies could also be tested more directly by comparing outcomes with a range of mechanisms. Mechanisms that are particularly complex or unrecognizable may be more difficult to use as embodied analogies, as the regular relationships among the mechanism’s parts will be harder to determine. Similarly, a mechanism that is irregular should not be able to be used as an embodied analogy and should yield no benefit to mathematical outcomes (beyond any impacts from other physical affordances). Future studies should test the relationship between mechanistic knowledge and mathematical strategies by comparing mechanisms that should be more easily used as analogies versus those that should not. Similarly, it would be of interest to investigate whether the skill of recognizing mechanisms (i.e., identifying the parts and operations involved in a system, and determining the underlying relations) can be taught to students such that they are able to independently and habitually apply this thinking to novel mechanisms, and whether this would lead to mathematical improvements.

Although we argue that mechanistic knowledge supports mathematical strategy use, it was not possible to conclusively establish the direction of this relationship as our Mechanistic Manipulation was not wholly successful (though again, it should be noted that similar results were found using several different criteria for categorizing participants as High-Mechanistic versus Low-Mechanistic). A large number of participants in the original Low-Mechanistic condition were still able to understand the robot’s mechanism after completing the task. Though no participants included in analyses reported prior experience with LEGO NXT robotics, the robot’s mechanism is relatively simple and similar to mechanisms regularly encountered in the real world (e.g., in vehicles), which likely made it easy for participants to discover on their own. Future studies should utilize a more novel mechanism that cannot be recognized without explicit instruction to determine whether mechanistic knowledge causes more complex mathematical strategy use.

The alternative exists that using mathematical strategies may lead to greater mechanistic knowledge, such that prior mathematical knowledge is used to make sense of a mechanism. For example, Schwartz, Martin, and Pfaffman (2005) asked students to determine whether a balance scale would fall to the left or right. In one condition, the balance scale was displayed such that weight and distance were discrete and easily measurable to promote mathematical strategies; in another condition, the balance scale was displayed such that weight and distance were continuous and difficult to measure. When 11-year-olds in the easy-to-measure condition were asked to explain their reasoning or to show their math, they solved the balance beam problems at a level typical of adults, suggesting that mathematical strategies helped them to understand the mechanisms of the balance beam’s movements. In the current study, participants may have used their mechanistic knowledge to generate mathematical strategies and inform their mathematical understanding of the robot (i.e., mechanism to math); or, they may have first discovered the mathematical patterns between their inputted motor rotations and the robot’s traveled distance and used that to understand the robot’s mechanism (i.e., math to mechanism); or, there may have been a constant conversation between their mechanistic knowledge and mathematical strategies, where discoveries about mechanistic and/or mathematical patterns were used to inform and revise their understanding of the other (i.e., a reciprocal mechanism and math relationship). However, we note that Silk’s (2011) experimental manipulation study also found that emphasizing mechanism during instruction helped middle-school students to think mathematically about robot motion planning, suggesting that the direction of mechanism to mathematics is particularly plausible in this context.

Another possibility is that the relationship between mechanistic knowledge and mathematical strategy use is confounded by general intelligence: people who are more intelligent are simply more likely to create complex navigation strategies and to understand the relations between parts of the robot. However, this seems implausible, as Paper Folding Test score (thought to correlate with general intelligence, e.g., Visser et al., 2006) was not a significant predictor of Maze Navigation Task strategy and did not appear to be driving strategy choice. Exposure to the robot may also act as another confound, such that participants who spent more time with the robot before the Maze Navigation Task were more likely to understand its mechanisms and to generate mathematical strategies. If this were the case, then participants in the original High-Mechanistic condition should have used more mathematical strategies than the original Low-Mechanistic condition, as the original High-Mechanistic condition had additional exposure time (approximately 5 min) with the robot during the Mechanistic Manipulation; however, there were no significant differences in strategy use using the Original Condition assignment. Future studies may still wish to control for exposure time by including another control condition in which participants are exposed to the robot for the same amount of time as the High-Mechanistic condition without mechanistic instruction.

## Conclusions

Many researchers have explored the role of physical manipulatives and embodied cognition in performance learning. In the current study, we show that a particular kind of reasoning is useful for capitalizing on manipulatives for building productive mathematical strategies during problem solving. Specifically, high mechanistic knowledge positively related to the frequency and complexity of mathematical problem-solving strategies. The current results suggest that emphasizing the underlying mechanisms of a problem may be helpful for students, allowing them to use manipulatives as an embodied analogy for abstract math concepts and to connect applied problems to the formal strategies learned in classrooms. Encouraging students to dissect mechanisms into their constituent parts and fully understand the relationships between those parts may emphasize the regular behaviors and patterns that underlie an applied problem context and are inherent to mathematics, fostering the application of mathematical problem-solving strategies.
